# Multiple Cold Tolerance Trait Phenotyping Reveals Shared Quantitative Trait Loci in *Oryza sativa*

**DOI:** 10.1186/s12284-020-00414-3

**Published:** 2020-08-14

**Authors:** Naoki Shimoyama, Melineeh Johnson, André Beaumont, Michael Schläppi

**Affiliations:** Department of Biological Sciences, Marquette University, Milwaukee, WI 53233 USA

**Keywords:** Abiotic stress, Chilling stress, Genome wide association study (GWAS), Golgi, Rice (*Oryza sativa* L.), Secretory pathway, Ubiquitination

## Abstract

**Background:**

Developing chilling tolerant accessions of domesticated Asian rice is a potential source of significant crop improvement. The uniquely chilling sensitive nature of the tropically originating *Oryza sativa* make it the most important cereal crop that can gain significantly from improved tolerance to low temperatures. However, mechanisms underlying this complex trait are not fully understood. *Oryza sativa* has two subspecies with different levels of chilling tolerance, *JAPONICA* and *INDICA*, providing an ideal tool to investigate mechanistic differences in the chilling stress tolerance responses within this important crop species.

**Results:**

The Rice Diversity Panel 1 (RDP1) was used to investigate a core set of *Oryza sativa* accessions. The tools available for this panel allowed for a comprehensive analysis of two chilling tolerance traits at multiple temperatures across a 354-cultivar subset of the RDP1. Chilling tolerance trait values were distributed as mostly subpopulation specific clusters of Tolerant, Intermediate, and Sensitive accessions. Genome-wide association study (GWAS) mapping approaches using all 354 accessions yielded a total of 245 quantitative trait loci (QTL), containing 178 unique QTL covering 25% of the rice genome, while 40 QTL were identified by multiple traits. QTL mappings using subsets of rice accession clusters yielded another 255 QTL, for a total of 500 QTL. The genes within these multiple trait QTL were analyzed for Gene Ontology (GO) term and potential pathway enrichments. Terms related to “carbohydrate biosynthesis”, “carbohydrate transmembrane transport”, “small molecule protein modification”, and “plasma membrane” were enriched from this list. Filtering was done to identify more likely candidate pathways involved in conferring chilling tolerance, resulting in enrichment of terms related to “Golgi apparatus”, “stress response”, “transmembrane transport”, and “signal transduction”.

**Conclusions:**

Taken together, these GO term clusters revealed a likely involvement of Golgi-mediated subcellular and extracellular vesicle and intracellular carbohydrate transport as a general cold stress tolerance response mechanism to achieve cell and metabolic homeostasis under chilling stress.

## Background

### Rising Global Food Demand Requires Significant Improvement in Abiotic Stress Tolerance

It was recently estimated that, by the year 2050, a 50% increase in food production is required to meet the needs of a growing global population (Food and Agriculture Organization of the United Nations (FAO) 2017). However, the rates of increase for major crops are falling significantly short to meet this demand. One possible method for increasing crop yields is to reduce losses caused by abiotic stress. Significant yield losses occur due to chilling stress every year (da Cruz et al. 2013; Singh et al. 2017). As global climate change continues to affect the predictability of seasonal temperatures, it will be important to significantly improve the resilience of our crops to aberrant cold snaps and unseasonably cold temperatures during critical stages of plant development, such as the young seedling stage.

### Rice as a Natural Model for Understanding Chilling Stress Tolerance Response Mechanisms

Plant chilling tolerance is a complex trait and its mechanisms have not yet been fully elucidated. Understanding cold stress tolerance response mechanisms is paramount to improve chilling tolerance of crop plants such as rice. Domesticated rice, *Oryza sativa* L., is a major food source for about half of the world population (McLean et al. 2013). However, current annual increases in rice production are not sufficient to meet the needs of our growing population. Unlike other staple crops, rice is uniquely susceptible to chilling stress due to its tropical and subtropical origins. Every year, chilling stress causes major yield loss in the rice crop (da Cruz et al. 2013). To eventually reduce such losses, our study aims to better understand cold stress tolerance response mechanisms in young seedlings, a critical stage for rice phenology.

We use domesticated rice as a model to investigate plant chilling tolerance (Schläppi et al. 2017; Liu et al. 2018; Shi et al. 2020). Rice is a useful tool for studying chilling tolerance due to its significant chilling sensitivity because of the tropical origins of its ancestors. The threshold temperature for chilling stress induced damage in rice is around 10 °C while other major crops such as winter wheat can withstand freezing temperatures (da Cruz et al. 2013). This feature indicates that compared to other major crops, improving chilling tolerance in domesticated rice may have one of the largest impacts on increasing crop yields. Additionally, due to artificial human selection, there are two distinct varietal groups within the rice species with significantly different chilling tolerance abilities (Shakiba et al. 2017; Liu et al. 2018). The *JAPONICA* subspecies, consisting of *temperate japonica*, *tropical japonica*, and *aromatic* subpopulations, is derived from varieties that were artificially selected and cultivated in temperate regions such as northern China, Japan, and Korea, where resistance to chilling temperatures was necessary. The *INDICA* subspecies, consisting of *aus* and various *indica* subpopulations, is derived from varieties that were artificially selected and cultivated in tropical and subtropical regions of southern and south eastern Asia, where selection for chilling tolerance traits was not necessary. These divergent varietal groups are extremely useful in identifying potential pathways associated with chilling tolerance. The fact that chilling tolerance within members of the *JAPONICA* subspecies was developed through artificial human selection instead of targeted genetic engineering raises the possibility that different chilling stress tolerance response mechanisms were selected in different rice accessions or entire subpopulations. Similarly, the large diversity found in the *INDICA* subspecies might provide insight into which chilling tolerance pathways were selected against in favor of other pathways that optimized other agricultural parameters.

### Genome-Wide Association Study (GWAS) Mapping of Chilling Tolerance Traits

Studying a wide range of genetically diverse population of rice cultivars provides us with a species-wide insight into chilling tolerance. By uncovering the mechanisms underlying the varietal group differences in chilling tolerance, it may be possible to improve chilling tolerance with limited genetic interventions by utilizing resistance systems already employed. To this end, the Rice Diversity Panel 1 (RDP1) developed as part of the Rice Diversity Project is a useful tool. It is a panel of 424 purified, homozygous accessions that are representative of the entire *O. sativa* species containing the five major subpopulations (*aromatic*, *aus*, *indica*, *temperate japonica*, and *tropical japonica*) and their admixed accessions (Eizenga et al. 2014; McCouch et al. 2016). This panel is additionally useful due to the genome-wide association study (GWAS) mapping pipeline developed by McCouch et al. 2016, which allows for in-house GWAS mapping even with limited computational resources. For these reasons, a subset of RDP1 accessions was used for phenotyping and GWAS mapping.

### Purpose of this Study

The major aim of this study was to help elucidating chilling stress tolerance response mechanisms in the generally cold sensitive crop species *Oryza sativa*. We show via genotype-based principal component analyses (PCA) combined with cold tolerance trait phenotyping analyses done at different temperatures that the RDP1 contains three distinct, mostly subpopulation-specific, clusters of accessions classified as chilling tolerant (Tolerant), intermediate chilling tolerant (Intermediate), and chilling sensitive (Sensitive). Through GWAS mapping using all accessions or accessions of the Tolerant and Sensitive clusters and accessions within clusters, we identified 500 QTL and a large number of QTL region-associated chilling tolerance candidate genes. We then used several filtering methods to reduce the number of candidate genes to identify the most probable chilling tolerance response pathways that are significantly altered between the chilling tolerant *JAPONICA* (*aromatic*; *temperate japonica*; *tropical japonica*) and chilling sensitive *INDICA* (*aus*; *indica*) subspecies.

## Results

### Chilling Tolerance Trait Phenotyping of RDP1 Accessions

**Fig. 1:**
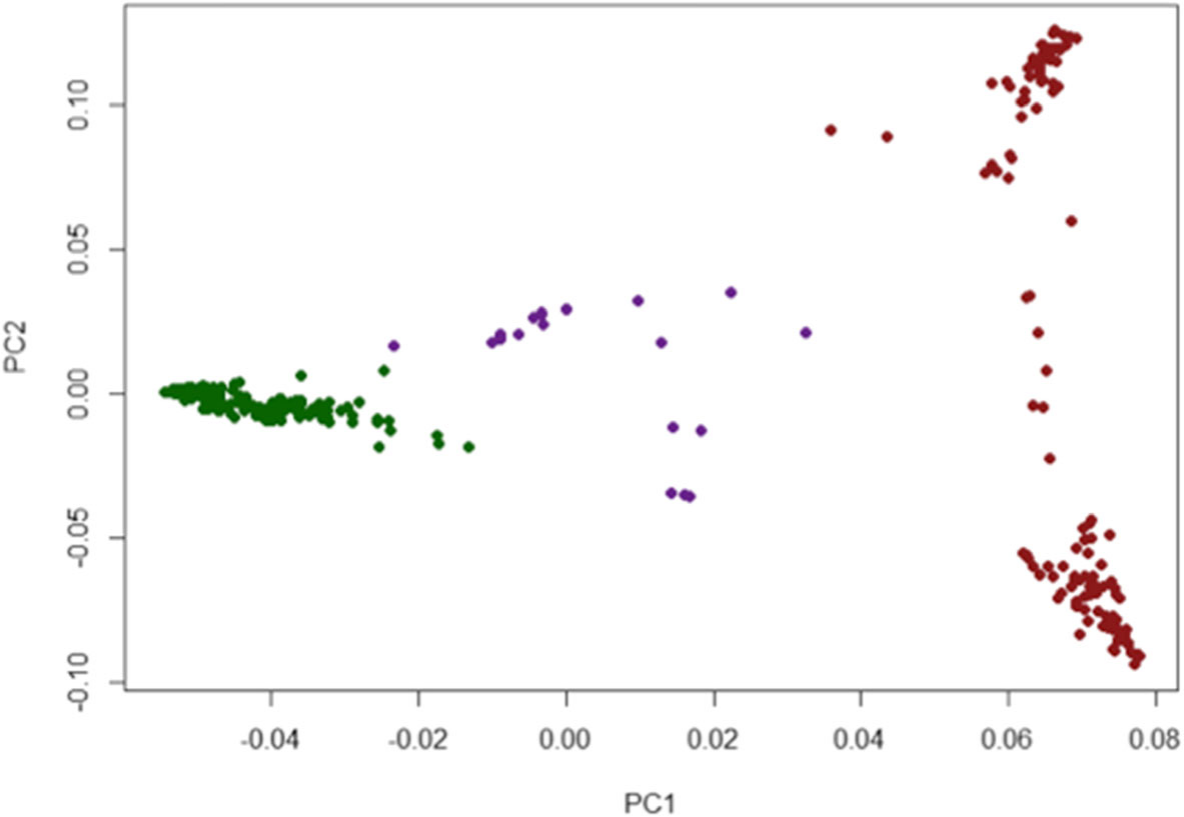
Principle Component Analysis (PCA) to illustrate genotype-based justification for rice accession subgroup clustering based on chilling tolerance phenotypes. PCA was calculated using the 700 K HDRA SNP profiles of 354 Rice Diversity Panel 1 accessions. Green, Cold Tolerant cluster (*temperate japonica*; *tropical japonica*, *japonica admixes*; 205 accessions). Purple, Intermediate Cold Tolerant cluster (*aromatic*; *indica*-*japonica* admixes; 20 accessions). Red, Cold Sensitive cluster (*aus*, various *indica*; *aus*-*indica* admixes; 129 accessions)

**Fig. 2:**
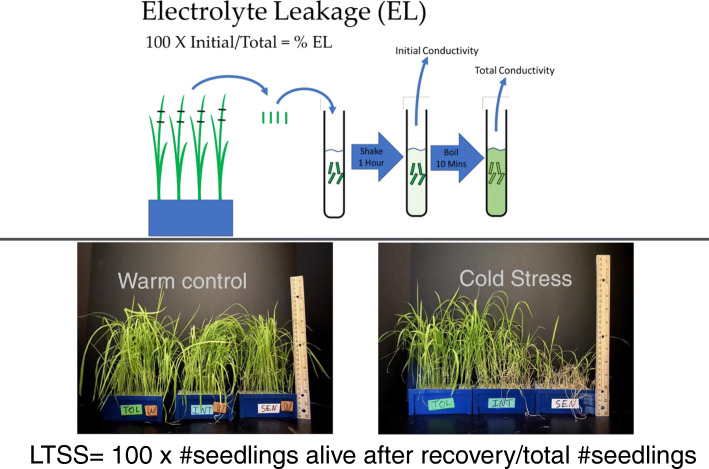
Electrolyte Leakage (EL) and Low-temperature Seedling Survivability (LTSS) phenotype analyses. Upper panel: EL assays were done using middle sections of leaves from two-week-old seedlings exposed continuously to 10 °C for 1 week. Lower panel: Left side, phenotype of three-week-old rice seedlings grown at warm (W) temperatures. Right side, phenotype of two-week-old seedlings exposed continuously to 10 °C for 1 week and after a one-week recovery period at warm temperatures. Most Col Tolerant (TOL) accessions are green and healthy looking. Most Cold Sensitive (SEN) accession are bleached and wrinkled. Intermediate (INT) Col Tolerant accessions have an intermediate phenotype

To better understand the natural variation that allows the *JAPONICA* subspecies of rice to be more chilling tolerant than the *INDICA* subspecies, multiple phenotypic analyses on a subset of Rice Diversity Panel 1 (RDP1; Eizenga et al. 2014) accessions were done at five different temperatures (4 °C, 8 °C, 10 °C, 12 °C, and 16 °C). The core subset of 354 out of 424 RDP1 accessions was chosen due to their consistently robust germination rates and abundant seed availability, and because their genotypes were included in the High-Density Rice Array (HDRA) containing 700,000 single nucleotide polymorphisms (SNPs; Chen et al. 2014). A principal component analysis using the SNP information showed that there was indeed considerable genetic variation that led to a clustering of the different subpopulations, which is a critical requirement for genome wide association studies (GWAS) and quantitative trait loci (QTL) mapping (Fig. 1). The rationale for choosing different chilling temperatures was to determine whether different stress tolerance pathways are active at different temperatures, or whether common pathways can be identified that are active at overlapping temperatures. The low-temperature seedling survivability (LTSS) phenotypic assay was chosen, because it was previously shown that at 10 °C, it significantly discriminated between accessions with different cold stress tolerance potentials (Schläppi et al. 2017). The electrolyte leakage (EL) phenotypic assay was chosen as a measure of plasma membrane integrity after cold temperature stress exposure, and it was previously shown that it can identify accessions with different cold stress tolerance potentials even when no differences in LTSS scores are observable (Shi et al. 2020). Phenotypic assays for EL and LTSS are shown in Fig. 2. Lastly, the median lethal chilling temperature (LT50), that is, the lethal temperature at which 50% of two-week-old seedlings die and 50% live after 1 week of cold exposure was calculated from LTSS scores to potentially identify additional chilling stress tolerance pathways at the threshold of survivability.

**Fig. 3:**
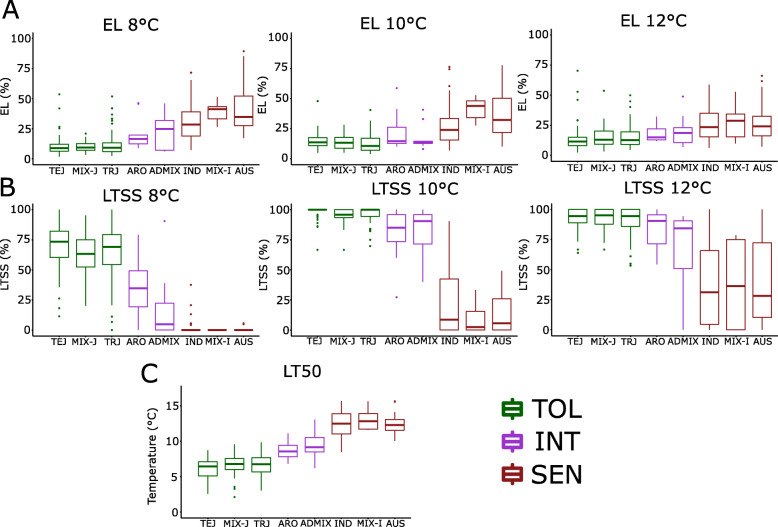
Cold tolerance trait analyses of eight rice subpopulations from the Rice Diversity Panel 1. Analyses were done at three chilling temperatures (8 °C, 10 °C, 12 °C) for Electrolyte Leakage (EL), Low-Temperature Seedling Survivability (LTSS), and Median Lethal Temperature (LT50). **a** LTSS. **b** EL. **c** LT50. TeJ, *temperate japonica*; MIX-J, admixed *japonica*; TRJ, *tropical japonica*; ARO, *aromatic*; ADMIX, admixed *aus*/*indica*-*japonica*; IND, *indica*; MIX-I, admixed *aus*-*indica*; AUS, *aus*. Three Cold Tolerance clusters are recognized: TOL, Cold Tolerant (green) cluster (TEJ, MIX-J, TRJ; 205 accessions); INT, Intermediate Cold Tolerant (purple) cluster (ARO, ADMIX; 20 accessions); SEN, Cold Sensitive (red) cluster (IND, MIX-I, AUS; 129 accessions)

Collectively, as shown in Fig. 3 for three temperatures (8 °C, 10 °C, and 12 °C), the *JAPONICA* and *INDICA* subspecies had significantly different LTSS, EL, and LT50 scores. Accessions within a particular subpopulation (*aromatic*, *aus*, *indica*, *temperate japonica*, *tropical japonica,* admixed *JAPONICA*, admixed *INDICA*, and admixed *INDICA/JAPONICA*) had similar chilling tolerance phenotypes, and they followed similar trajectories at every temperature tested. Interestingly, while most members of the *JAPONICA* subspecies were more chilling tolerant than members of the *INDICA* subspecies, *aromatic* accessions which are typically classified as part of the *JAPONICA* subspecies had chilling tolerance phenotypes that were more similar to admixed *INDICA/JAPONICA* than to other *JAPONICA* relatives, most likely because genotypically they also clustered with admixed varieties (Fig. 1). In agreement with our results, it was previously observed that *aromatic* accessions clustered with admixed instead of *temperate* or *tropical japonica* accessions (Wang et al. 2018). For this reason, *aromatic* and admixed *INDICA/JAPONICA* accessions were grouped into a third “Intermediate Cold Tolerant” (Intermediate) cluster, distinct from the “Cold Tolerant” (Tolerant; *temperate japonica*, *tropical japonica*, and admixed *JAPONICA*) and “Cold Sensitive” (Sensitive; *aus*, *indica*, and admixed *INDICA*) clusters. Admixed *JAPONICA* and admixed *INDICA* accessions predictably had phenotypes in accordance with their *JAPONICA* and *INDICA* mixed genotypes, respectively. However, it is interesting to note that admixed *JAPONICA* accessions were generally a bit less chilling tolerant than either *temperate* or *tropical japonica* accession while admixed *INDICA* accessions were generally a bit more chilling sensitive than either *aus* or *indica* accessions (Fig. 3). The three “chilling tolerance clusters” were used for multiple chilling tolerance trait analyses, which was the basis for subsequent GWAS mapping.

### Summary of Percent Electrolyte Leakage (EL) Results

**Fig. 4:**
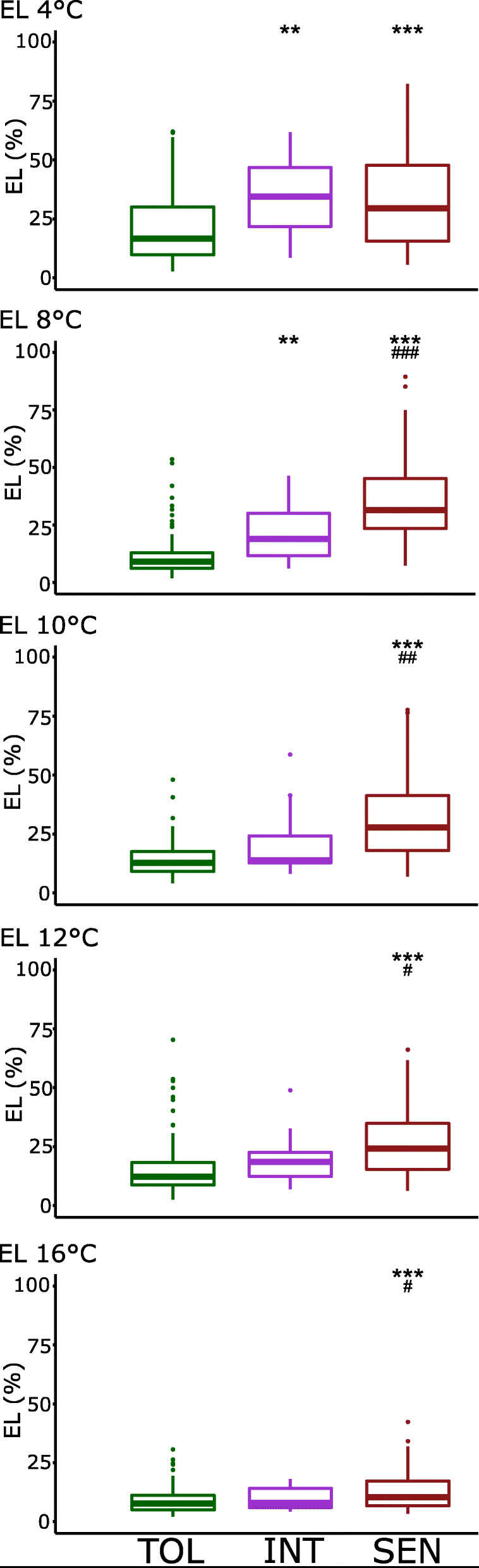
Box plots of mean Electrolyte Leakage (EL) values at different chilling temperatures for rice accessions in each cold tolerance cluster. Significant differences compared to the Cold Tolerant (TOL) cluster at all temperatures is labeled by ^*^. Significant differences compared to the Intermediate (INT) Col Tolerant cluster is labeled by ^**#**^. SEN, Cold Sensitive Cluster. ^***^p < 0.001; ^**^p < 0.01; ^*^p < 0.05. ^**###**^*p* < 0.001; ^**##**^*p* < 0.01; ^**#**^*p* < 0.05

The percent EL was determined for each cultivar as a measure of plasma membrane integrity after cold temperature stress exposure, with the general expectation that cold sensitive accessions will release more electrolytes (high EL scores) from leaf tissues than cold tolerant accession (low EL scores), due to a higher incidence of membrane lesions after cold exposure. The mean percent EL for Tolerant, Intermediate, and Sensitive clusters generally followed this expectation (Fig. 4). For the Tolerant cluster, the mean percent EL at the five temperatures ranged from 8.7 to 20.9%; for the Intermediate cluster, 9.4 to 34.3%; and for the Sensitive cluster, 12.8 to 35.4%. At each temperature, mean EL values were significantly lower for the Tolerant cluster than for the Sensitive clusters. Mean EL values were also significantly lower for the Tolerant cluster than the Intermediate cluster at 4 °C and 8 °C, however, there were no significant differences between 10 and 16 °C. This indicates that below 10 °C, the Intermediate cluster shifts from a more tolerant to a more sensitive EL phenotype. For the Sensitive cluster, similar EL values at 4 °C and 8 °C suggests that 8 °C is a threshold temperature for Sensitive accessions below which lower temperatures do not significantly increase membrane lesions any more. Taken together, these EL results suggest that Tolerant, Intermediate, and Sensitive clusters have different low-temperature thresholds at which maximum membrane lesions occur, and that each cluster has a different low-temperature buffering range for maintenance of membrane integrity.

### Summary of Low-Temperature Seedling Survivability (LTSS) Results

**Fig. 5:**
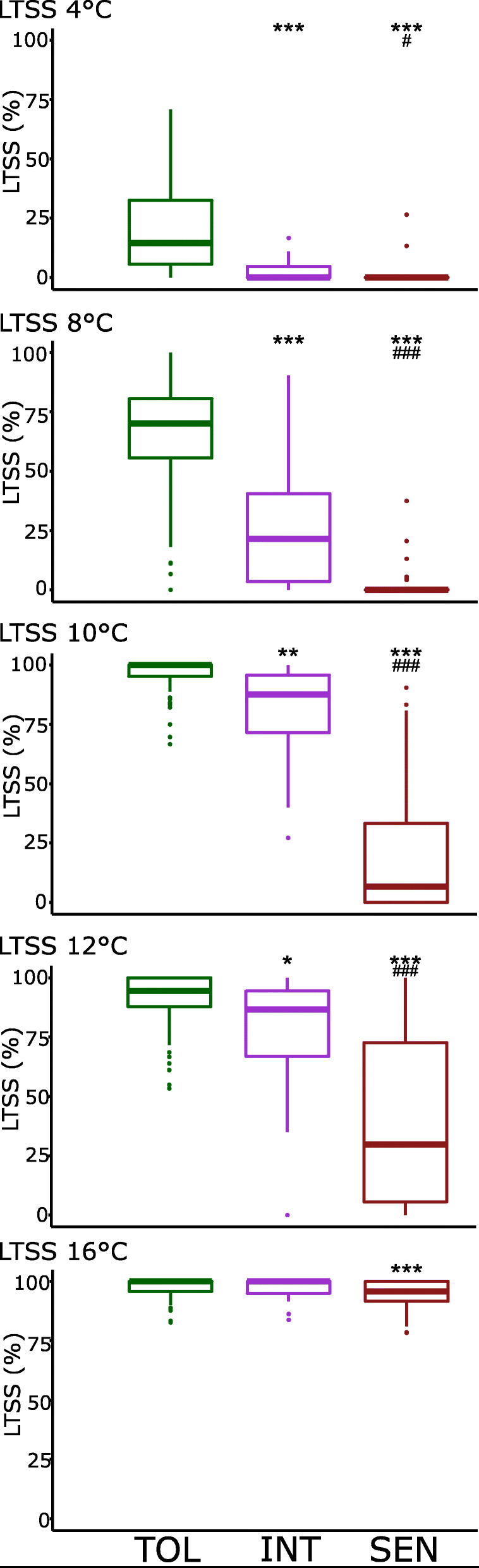
Box plots of mean Low-Temperature Seedling Survivability (LTSS) values at different chilling temperatures for rice accessions in each cold tolerance cluster. Significant differences compared to the Cold Tolerant (TOL) cluster at all temperatures is labeled by ^*^. Significant differences compared to the Intermediate (INT) Col Tolerant cluster is labeled by ^**#**^. SEN, Cold Sensitive Cluster. ^***^p < 0.001; ^**^p < 0.01; ^*^p < 0.05. ^**###**^*p* < 0.001; ^**##**^*p* < 0.01; ^**#**^*p* < 0.05

The percent LTSS was determined as a measure of survival of two-week-old seedlings after 1 week of continuous low-temperature stress exposure. The phenotype of plants after a 7-day recovery period from cold stress is shown in Fig. 2: recovered seedlings were alive, green, and healthy looking while dead seedlings were bleached and wilted. Our expectation was that the Tolerant cluster will have significantly more accessions with high LTSS scores than the Intermediate and Sensitive clusters. The mean percent LTSS for Tolerant, Intermediate, and Sensitive clusters generally followed this expectation (Fig. 5). For the Tolerant cluster, the mean percent LTSS at the five temperatures ranged from 20.1 to 98.2%; for the Intermediate cluster, 3.0 to 96.2%; and for the Sensitive cluster, 0.3 to 94.9%. At each temperature, mean LTSS scores were significantly lower for the Sensitive cluster than for the other two clusters, and except for 16 °C, mean LTSS values were significantly higher for the Tolerant cluster than the Intermediate cluster. Between 4 and 10 °C, mean LTSS values for the Sensitive cluster were very similar, indicating that 10 °C might be a threshold temperature for Sensitive accessions below which lower temperatures do not significantly decrease seedling survivability, which is similar to what was observed for the EL phenotype. Between 8 and 10 °C, LTSS values for the Intermediate cluster shifted from a more tolerant to a more sensitive phenotype, which is also similar to what was observed for the EL phenotype, however, an apparent threshold for survivability was reached between 4 and 8 °C. The somewhat different temperature threshold buffering ranges for mean EL and LTSS scores suggests that there may not be a linear correlation between membrane integrity and seedling survivability.

### Correlation between LTSS and EL at Different Low Temperatures

**Fig. 6:**
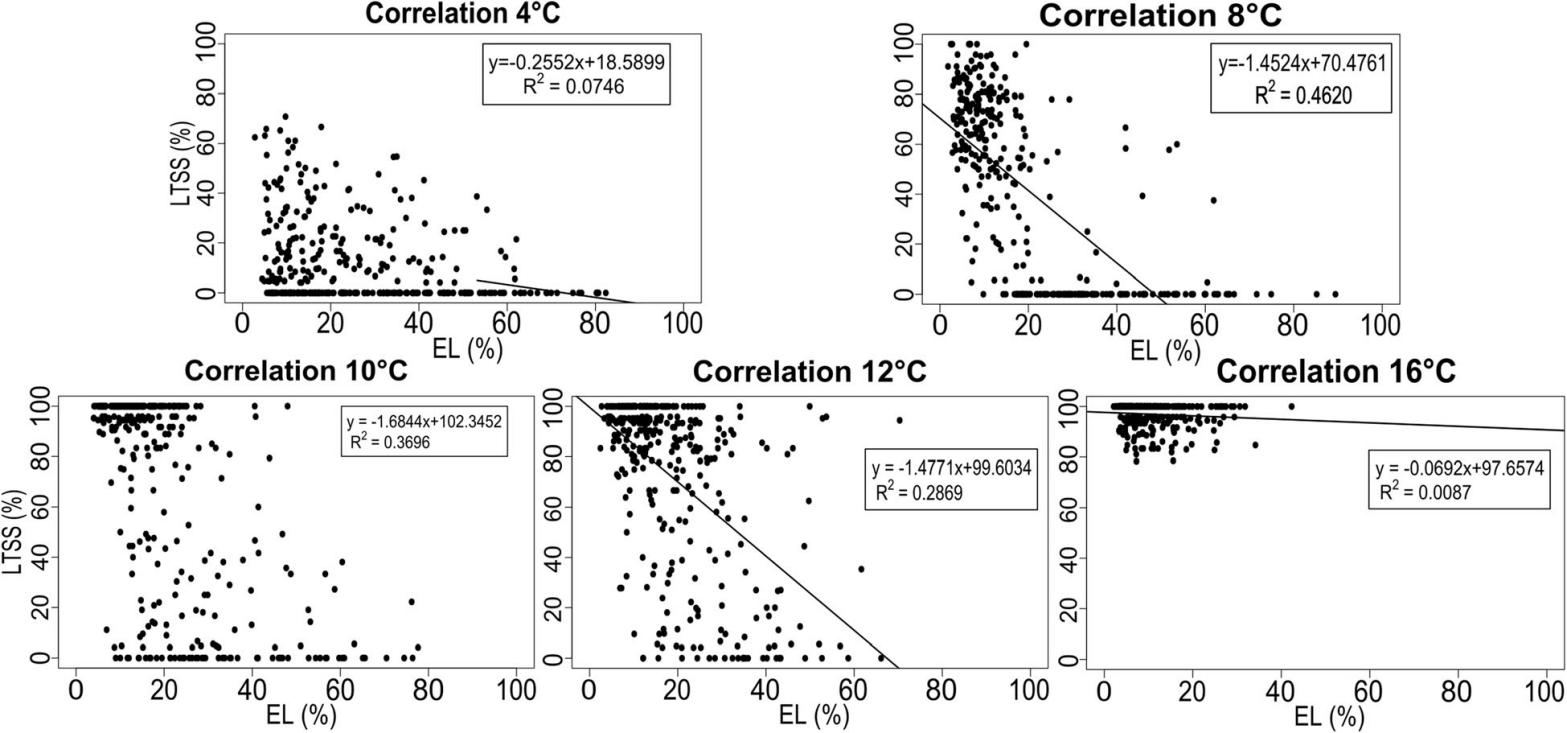
Correlation of Electrolyte Leakage (EL, x axis) and Low-Temperature Seedling Survivability (LTSS, y axis) phenotypes measured at the five different chilling temperatures of 4 °C, 8 °C, 10 °C, 12 °C, and 16 °C. Trendlines and *R*^2^ correlation coefficients were calculated using a linear curve fit

Percent EL and LTSS phenotypes of the Intermediate cluster were similar to those of the Tolerant and Sensitive clusters at high and low temperatures, respectively, but at different threshold temperatures (Figs. 4 and 5). This suggests that the EL phenotype, measured immediately after 1 week of low-temperature stress exposure, might not accurately predict survival rates 1 week after recovery at growth promoting temperatures. Therefore, correlations between EL and LTSS were done to determine how effectively EL scores can be used to predict LTSS. Interestingly, linear correlation coefficients varied between the five temperatures (Fig. 6). The best correlation was observed at 8 °C (*R*^*2*^ = 0.4620), followed by 10 °C (*R*^*2*^ = 0.3696) and 12 °C (*R*^*2*^ = 0.2869), while there was no correlation at the extreme ends of 4 °C (*R*^*2*^ = 0.0746) and 16 °C (*R*^*2*^ = 0.0098). This indicates that at 8 °C ± 1 °C, the degree of chilling temperature induced membrane damage correlates well with subsequent probability for survival. That is, less membrane damage predicts higher survival rates, however, even at 8 °C, there were several outliers with little membrane damage and low survival rates, and several outliers with a high degree of membrane damage and high survival rates (Fig. 6). This suggests that for some accessions, membrane damage sustained during cold exposure is not the main cause for low survival rates, indicating that physiological or metabolic events during the recovery phase affect survival rates. In contrast, even though significant membrane damage occurs during cold exposure, some accessions can deal with this damage during the recovery phase, leading to increased survival rates.

### Summary of Median Lethal Chilling Temperature (LT50) Results

**Fig. 7:**
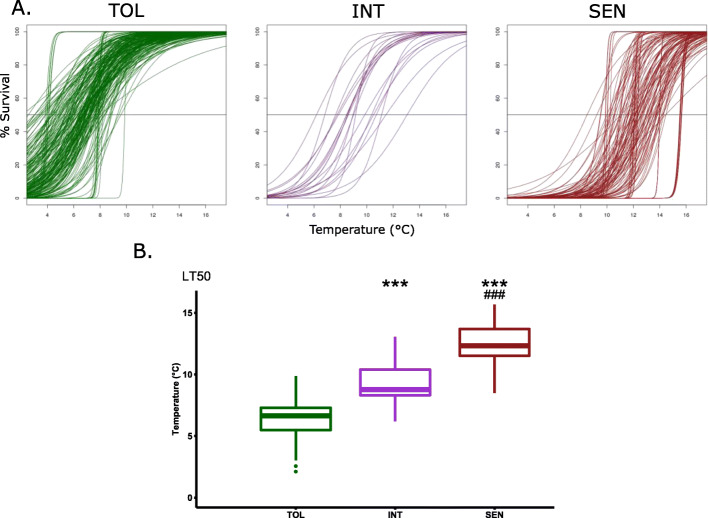
Fitted Low-temperature Seedling Survivability (LTSS) curves at different temperatures and Median Lethal Temperatures (LT50). **a** LTSS values (% Survival) obtained at 4 °C, 8 °C, 10 °C, 12 °C, and 16 °C were fit to a sigmoidal curve using a generalized linear model in *R*. The line at 50% Survival indicates the temperature at which 50% of the accessions lived and 50% of the accessions died, generating LT50 values. **b** Box plots of mean LT50 values for the three Cold Tolerance clusters. TOL, Cold Tolerant cluster; INT, Intermediate Cold Tolerant cluster; SEN, Cold Sensitive Cluster. Significant *p* values in comparison to the TOL cluster are labeled by ^*^. Significant *p* values in comparison to the INT cluster are labeled by ^**#**^. ^***^p < 0.001; ^**^p < 0.01; ^*^p < 0.05. ^**###**^*p* < 0.001; ^**##**^*p* < 0.01; ^**#**^*p* < 0.05

Our data show that different “cold tolerance clusters” (Figs. 4 and 5) as well as different subgroups (Fig. 3) had different “tipping points” where EL or LTSS scores changed dramatically. To include these tipping points as an additional parameter for GWAS analyses, we calculated LT50 values from LTSS data for each accession at which 50% of seedlings died and 50% survived. Using *R*, LTSS curves were generated by plotting median LTSS rates at 4 °C, 8 °C, 10 °C, 12 °C, and 16 °C to identify LT50 for each accession (Fig. 7a). The curves were clustered for Tolerant, Intermediate, and Sensitive accessions, and median LT50 were calculated for each cluster (Fig. 7b). The survival curves and median LT50 values for each tolerance cluster generally followed the patterns observed for LTSS, further supporting our classification of *aromatic* accessions as an Intermediate cluster.

### Genome-Wide Association Study (GWAS)-Based Mapping of EL, LTSS, and LT50 QTL

**Fig. 8:**
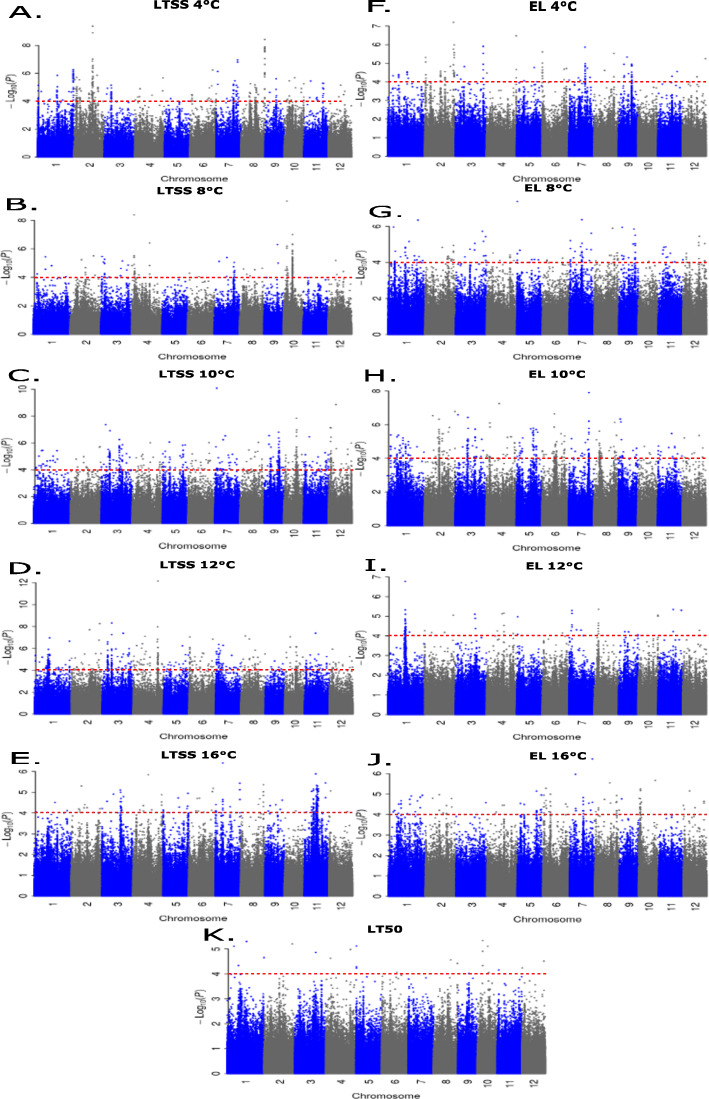
Manhattan plots showing the probability [−log10(*p*)] of 700,000 single nucleotide polymorphisms (SNPs) within 354 rice accessions to be associated with 11 cold tolerance traits: LTSS, Low-Temperature Seedling Survivability, measured at 4 °C, 8 °C, 10 °C, 12 °C, and 16 °C (Fig. 5); EL, Electrolyte Leakage, measured at 4 °C, 8 °C, 10 °C, 12 °C, and 16 °C (Fig. 4); LT50, Median Lethal Temperature (Fig. 7). The red dotted line indicates that SNPs with values of -log10(*p*) > 4 are significant and were used for genome-wide association study (GWAS)-based mapping of cold tolerance quantitative trait loci (QTL)

From the core subset of 354 RDP1 accessions, EL, LTSS, and LT50 data were used to perform GWAS to identify significantly trait associated SNPs. We applied a publicly available GWAS pipeline (McCouch et al. 2016) that accounted for population structure of the rice accessions using a kinship matrix created by the efficient mixed-model association eXpedited (EMMAX) approach (Kang et al. 2010), which determined ancestry or cryptic relatedness between individual genotyping markers. GWAS-mapping pipeline produced quantile-quantile (Q-Q) plots and Manhattan plots for each cold tolerance trait are shown in Fig. S1 and Fig. 8, respectively. Although several Q-Q plots appeared to indicate inflation in some of the phenotypes, our plots had similar trends as those of the original mapping results reported in the study that established the RDP1 mapping pipeline (McCouch et al. 2016). Therefore, based on Q-Q plots a *p*-value cutoff for significant SNPs was determined to be at -log10(*p*) > 4, and QTL were identified as chromosomal sites containing 3 significant SNPs within a one million base pair (Mbp) region.

**Fig. 9:**
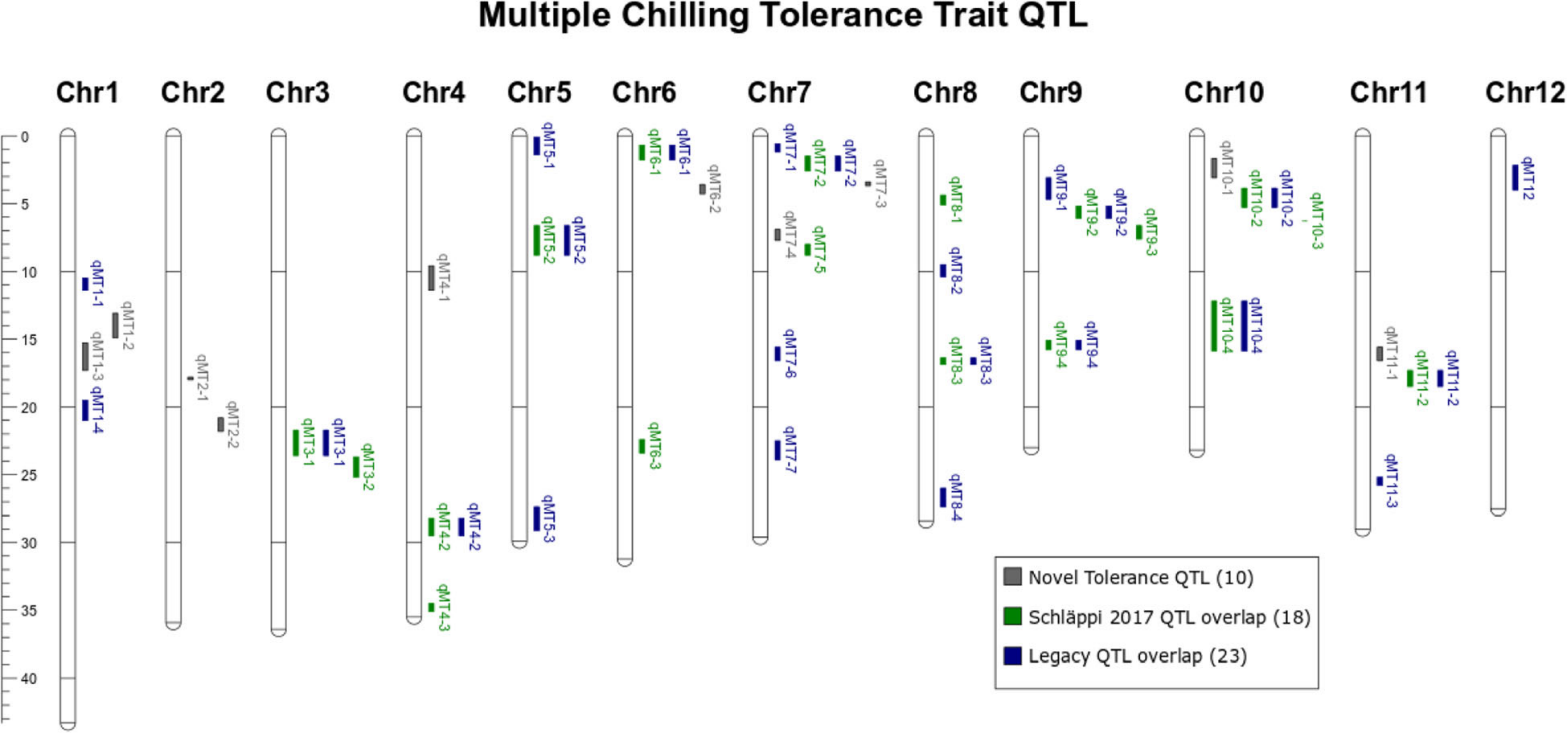
Genomic locations of 40 Multiple-Trait QTL (*qMT*). Bars show the genomic regions of *qMT* QTL covering at least two of the following individual trait QTL: Electrolyte Leakage (EL) measured at 4 °C, 8 °C, 10 °C, 12 °C, or 16 °C (Fig. 4); Low-Temperature Seedling Survivability (LTSS) measured at 4 °C, 8 °C, 10 °C, 12 °C, or 16 °C (Fig. 5); and Median Lethal Temperature (LT50, Fig. 7). Grey bars show novel seedling stage cold tolerance QTL identified in this study, while green bars show *qMT* overlapping with various seedling stage cold tolerance QTL identified by Schläppi et al. 2017, and blue bars show *qMT* overlapping with legacy seedling stage cold tolerance QTL identified by Andaya and Mackill 2003; Liu et al. 2013; Ma et al. 2015; Lv et al. 2016; and Wang et al. 2016

Across the three traits and five temperatures, 245 total QTL were identified. The LTSS trait yielded 150 QTL, the EL trait 92 QTL, and the LT50 trait 3 QTL (Table S1-S7). Temperature also affected the number of QTL identified. The moderate chilling temperatures of 10 °C and 12 °C yielded 83 and 62 QTL covering 11.6% and 8.9% of the rice genome, respectively, while 4 °C, 8 °C, and 16 °C yielded 38, 29, and 30 QTL respectively (Table S1-S5). Many of the 245 QTL were found in overlapping chromosomal regions, some of which were longer than 1 Mbp (Fig. 9; Fig. S2). Taken together, we identified 178 unique QTL, consisting of 40 multiple-trait QTL (*qMT*) and 138 single-trait QTL (*qE* for *qEL*, and *qL* for *qLTSS*) (Table S7), covering approximately 25% of the rice genome.

Using different cold tolerance clusters, additional GWAS-based QTL mappings were done. To identify QTL that control chilling tolerance in accessions of the Tolerant and Sensitive clusters, the Intermediate cluster was removed and GWAS mappings were done for the three high EL-LTSS-correlation temperatures of 8 °C, 10 °C, and 12 °C (Table S8). GWAS mapping was also done within the Tolerant and Sensitive clusters to identify QTL correlating with phenotypic variability within each subspecies for the three high EL-LTSS-correlation temperatures of 8 °C, 10 °C, and 12 °C (Table S9). The Tolerant versus Sensitive (TvS) GWAS mapping resulted in 219 individual QTL: 68 EL QTL, 150 LTSS QTL, and 1 LT50 QTL. The within-subspecies GWAS mapping resulted in 36 individual QTL: Mapping within the Sensitive cluster yielded 33 QTL consisting of 3 EL QTL, 29 LTSS QTL, and 1 LT50 QTL; and mapping within the Tolerant cluster yielded 3 QTL consisting of 2 EL QTL and 1 LTSS QTL. This result is consistent with the larger genetic variation within Sensitive (Fig. 1, red cluster) than Tolerant (Fig. 1, green cluster) accessions.

Within the 178 unique QTL regions obtained using all 354 accessions, 14,710 genes were identified, which is more than 25% of the total number of annotated genes reported by the Rice Genome Annotation Project (Kawahara et al. 2013). Since a major focus of our GWAS analysis was to identify general cold tolerance genes with natural variation between chilling tolerant and chilling sensitive rice accessions and to reduce the list of potential candidates, we interrogated the 40 *qMT* QTL regions for chilling tolerance candidate genes (Table 1). There were 30 QTL that overlapped with previously published cold tolerance QTL (Andaya and Mackill 2003; Liu et al. 2013; Ma et al. 2015; Wang et al. 2016; Lv et al. 2016; Schläppi et al. 2017). Genes found in the *qMT* regions are hypothesized to be involved in either multiple traits (EL, LTSS, LT50) or in helping with improving those traits at more than one temperature. This reduced the total number of genes to be analyzed from 14,710 to 6065 genes associated with the 40 *qMT* regions, which were further analyzed using gene function and Gene Ontology (GO) term annotations.

**Table 1:** Table of Multiple Trait QTL

QTL	Chr	Start	End	Mapping Phenotypes	Cluster Overlap	Peak LOD	Peak SNP gene	Published QTL
*qMT1–1*	1	10,527,625	11,389,874	E10 + L10	TvS	5.444609		*Locus2* ^*a*^
*qMT1–2*	1	13,105,151	14,895,303	E10 + E16 + L12	TvS	4.769976		
*qMT1–3*	1	15,272,806	17,301,559	E10 + L12	TvS	5.906907		
*qMT1–4*	1	19,518,259	20,986,910	E8 + E12	TvS	6.768674		*Locus4* ^*a*^*, qCTS1–3* ^*c*^
*qMT2–1*	2	17,820,486	18,028,915	L4 + L8		4.680601	LOC_Os02g30270	
*qMT2–2*	2	20,805,063	21,772,969	E10 + L4	TvS	7.292885		
*qMT3–1*	3	21,728,962	23,622,225	E10 + E12 + L8 + L10 + L12 + L16	S + TvS	6.272784		*qCTS3–11* ^*c*^
*qMT3–2*	3	23,729,655	25,203,390	E10 + L10 + L12	S + TvS	7.380266		*qLTSS3–3/qLTS3–2* ^*b*^
*qMT4–1*	4	9,599,400	11,394,804	L10 + L12	TvS	6.484325		
*qMT4–2*	4	28,197,218	29,473,755	E10 + L12	TvS	12.14515		*Locus47* ^*a*^*, qLTSS4–2* ^*b*^*, qCTS4–3* ^*e*^
*qMT4–3*	4	34,467,694	35,089,971	E4 + L10	S + TvS	6.47215	LOC_Os04g58780	*qLTSS4–3/qLTS4* ^*b*^
*qMT5–1*	5	69,986	1,409,302	E8 + L10 + L16 + LT50	TvS	7.38114		*Locus51* ^*a*^
*qMT5–2*	5	6,600,746	8,783,967	E10 + L10 + L12	TvS	6.324189		*qLTS5* ^*b*^*, qCTS5–3* ^*c*^
*qMT5–3*	5	27,413,208	29,075,588	E16 + L16		4.955528	LOC_Os05g48960	*Locus56* ^*a*^
*qMT6–1*	6	690,949	1,804,604	E16 + L12	S + TvS	7.250662	LOC_Os06g02710	*Locus57* ^*a*^*, qPGC6–1* ^*b*^
*qMT6–2*	6	3,567,142	4,288,870	E16 + L12	TvS	5.286129	LOC_Os06g07420	
*qMT6–3*	6	22,367,941	23,364,932	E10 + L4 + L12		5.466931		*qLTG6* ^*b*^
*qMT7–1*	7	592,074	1,198,046	L12 + L16	TvS	7.1194		*qCTS7–1* ^*c*^
*qMT7–2*	7	1,452,372	2,559,101	E8 + L10	S + TvS	10.08297		*Locus69* ^*a*^*, qLTSS7–1* ^*b*^
*qMT7–3*	7	3,402,347	3,683,917	E12 + L12	TvS	6.412739	LOC_Os07g07240	
*qMT7–4*	7	6,870,947	7,663,360	E16 + L12	TvS	5.970675	LOC_Os07g12330	
*qMT7–5*	7	7,997,910	8,776,532	L12 + L16	TvS	6.408		*qLTSS7–2* ^*b*^
*qMT7–6*	7	15,627,934	16,632,053	E16 + L10	S + TvS	5.094292		*Locus75* ^*a*^*, qCTS7* ^*d*^
*qMT7–7*	7	22,505,330	23,930,131	E10 + L8	TvS	7.906038		*qCTS7–4* ^*c*^
*qMT8–1*	8	4,409,763	5,060,164	E12 + L12	TvS	7.134518		*qLSS8–1* ^*b*^
*qMT8–2*	8	9,468,694	10,399,998	L4 + L12	TvS	7.793283		*qCTS8–2* ^*c*^
*qMT8–3*	8	16,369,660	16,896,911	L4 + L12	TvS	6.393616	LOC_Os08g27240	*Locus84* ^*a*^*, qLTSS8–2/qLTS8* ^*b*^
*qMT8–4*	8	26,014,290	27,371,604	E8 + E10 + L16	TvS	5.37007	LOC_Os08g41830	*Locus87* ^*a*^*, COLD2*^*f*^
*qMT9–1*	9	3,149,137	4,733,475	E8 + E10	TvS	6.185335		*Locus91* ^*a*^*, qCTS9–3/qCTS9–4/qCTS9–5* ^*c*^
*qMT9–2*	9	5,186,978	6,076,169	E8 + E10	TvS	8.264567		*Locus93* ^*a*^*, qPGCG9–1* ^*b*^
*qMT9–3*	9	6,569,358	7,630,726	E4 + L10	TvS	5.145748		*qPGCG9–1* ^*b*^
*qMT9–4*	9	15,055,972	15,835,730	E4 + L10	TvS	5.000415	LOC_Os09g25220	*Locus97* ^*a*^*, qLTG-9* ^*d*^*, qPGCG9–2/qLTSS9–2* ^*b*^
*qMT10–1*	10	1,740,269	3,057,764	E16 + L10	TvS	6.125558		
*qMT10–2*	10	3,909,466	5,322,884	L8 + L10	S + TvS	10.35381		*Locus101* ^*a*^*, qLTSS10–1* ^*b*^
*qMT10–3*	10	6,299,787	6,300,600	L12 + LT50	TvS	7.180984	LOC_Os10g11354	*qLTSS10–1* ^*b*^
*qMT10–4*	10	12,242,058	15,899,908	L10 + L12 + LT50	S + TvS	7.843522		*Locus104* ^*a*^*, qLTSS10–2* ^*b*^
*qMT11–1*	11	15,593,585	16,583,082	L12 + L16		5.455106	LOC_Os11g28184	
*qMT11–2*	11	17,316,314	18,487,194	E10 + L12	S + TvS	5.02026	LOC_Os11g31620	*Locus111* ^*a*^*, qLTSS11–3* ^*b*^*, qCTS11–5* ^*c*^
*qMT11–3*	11	25,159,572	25,809,234	L10 + L12		5.266234		*Locus116* ^*a*^*, qCTS11–10* ^*c*^
*qMT12*	12	2,192,895	4,043,986	E8 + L10 + L12	S + TvS	8.710121	LOC_Os12g06270	*Locus121* ^*a*^

*qMT* Multi-Trait QTL containing overlapping individual QTL mapped with cold tolerance traits

*E* Electrolyte Leakage

*L* Low-Temperature Seedling Survivability

LT50: median low temperature of seedling survival

4, 8, 10, 12, 16: 4 °C, 8 °C, 10 °C, 12 °C, 16 °C

TvS: Tolerant cluster vs Sensitive cluster QTL overlapping with *qMT* QTL

*T* Tolerant cluster QTL overlapping with *qMT* QTL

*S* Sensitive cluster QTL overlapping with *qMT* QTL

LOD: -log10(*p*) values of peak SNPs within *qMT* QTL

Peak SNP gene: gene within a *qMT* QTL containing a peak SNP

^a^Lv et al. 2016

^b^Schläppi et al. 2017

^c^Wang et al. 2016

^d^Liu et al. 2013

^e^Andaya and Mackill 2003

^f^Ma et al. 2015

To identify cold tolerance candidate genes within the *qMT* QTL, annotated genes that contained significant SNPs (*p* < 10^− 4^) within their genomic regions were selected, yielding a list of 220 genes. Significant SNPs with expected percentages of major alleles and direction of allele effect on chilling tolerance trait were used to further filter the gene list. That is, significant SNPs with a major allele frequency at least as much as the percentage of accessions of the Tolerant cluster (205 out of 354 accessions = 58%) and with an allele effect positively correlating with the measured chilling tolerance trait (to decrease EL and to increase LTSS) were selected, yielding a list of 81 candidate genes. Those genes were then used for GO term enrichment analysis and compared to the larger gene list to narrow down possible cold tolerance response pathways. Furthermore, since GWAS-based QTL mapping was also done using accessions from between and within the major cold Tolerant and Sensitive clusters, 35 *qMT* QTL regions with at least one single QTL using these clusters were identified and used for targeted GO enrichment analysis (Table 1). In addition to using a kinship matrix, this analysis was done to reduce the potential influence of population structure in defining significant regions. Thus, the rationale for using regions that were highlighted in mappings within clusters was to account for a potential population structure bias arising from only the differences in variability between the Sensitive and Tolerant clusters. Those 35 *qMT* QTL were further filtered for genes containing significant SNPs, which resulted in 207 genes that were separately used for GO term enrichment analysis.

### GO Term Analyses of all Genes within Multi-Trait (*qMT*) QTL

The *qMT* QTL gene list was purged of annotated transposable elements resulting in 4256 genes which were analyzed for GO term enrichment. GO term annotations were obtained for 2483 of the 4256 genes, and 257 GO terms were found to be enriched (*p* < 0.01), and those relevant to plant chilling tolerance were grouped into chilling stress relevant clusters. Within the GO term for *Biological Process*, three major clusters were identified for “carbohydrate derivative biosynthesis”, “protein modification by small protein conjugation or removal”, “carbohydrate transmembrane transport”, and “hydrogen peroxide mediated signaling pathway” (Fig. S3). Within the GO term for *Cell Component*, there were only two clusters: “transferase complex” and “intrinsic component of plasma membrane” (Fig. S4). Within the GO term for *Molecular Function*, numerous clusters were identified including: “ADP binding”, “carbohydrate transmembrane transporter activity”, “oxidoreductase activity”, “thiol-dependent ubiquitinyl hydrolase activity”, and “ubiquitin-like protein transferase activity” (Fig. S5). These terms suggest possible associations with chilling tolerance mechanisms and can be used to select pools of genes for further analysis. These genes are expected to be involved in metabolic homeostasis, membrane composition, ubiquitination, oxidative stress, and signaling pathways.

### GO Term Analysis of Filtered Gene List from *qMT* QTL

The filtered list of 81 genes containing significant SNPs was also purged of annotated transposable elements, reducing it to 71 genes for GO term enrichment analysis. Due to the low gene number, significance of enrichment was set to *p* < 0.05. GO term annotations were obtained for 45 genes and 10 terms were enriched. In *Biological Process*, “cellular response to stress” and “transmembrane transport” were enriched (Fig. S6). In *Cell Component*, “Golgi membrane” and “extracellular region” were enriched (Fig. S7). In *Molecular Function*, “molecular transducer activity” and “exonuclease activity” were enriched (Fig. S8). These enrichment clusters suggest likely interaction of genes involved in vesicle transport and secretion and nucleic acid processing to confer cold stress tolerance responses. Enrichment for cellular response to stress and Golgi membrane suggest that these filtered genes may be involved in modifications and transport of membranes and membrane-bound proteins.

### GO Term Analyses of Significant Genes within Multiple-Trait and Cold Tolerant & Cold Sensitive Cluster QTL

Genes within the 35 *qMT* QTL that overlapped with cold Tolerant and cold Sensitive cluster-specific QTL (Table 1) were separately investigated to identify enriched GO terms. Out of 67 input genes, the full filtering protocol resulted in only three enriched (*p* < 0.05) GO terms: “exonuclease activity”, “cytoplasmic part”, and “extracellular region”. As term enrichment map visualizations were not possible with so few terms, an assessment was done on a larger list of genes from an intermediate filtering protocol. The intermediate filtering of only genes with significant SNPs resulted in 159 non-transposable element genes with significant SNPs from the 35 *qMT* and Tolerant & Sensitive Cluster QTL. The terms that were enriched at this stage were related to “ubiquitination”, “plant growth”, and “nucleotide binding” (Fig. S9, S10 and S11).

## Discussion

### Chilling Tolerance Phenotype of each Cluster is Differentially Affected by Stress Severity

The percent low-temperature seedling survivability (LTSS) and percent electrolyte leakage (EL) means of different rice accessions at different chilling temperatures generally followed an expected trajectory based on their subpopulation structure, including the recent chilling tolerance reevaluation of *aromatic* and admixed accessions (Wang et al. 2018). That is, *temperate* and *tropical japonica* accessions and admixed *japonicas*, comprising the more chilling tolerant *JAPONICA* subspecies, generally had lower EL and higher LTSS scores than *aus* and *indica* accessions and *aus*/*indica* admixes, comprising the more chilling sensitive *INDICA* subspecies (Fig. 3). It is important to note that due to artificial human selection and geographic regions of cultivation, the *JAPONICA* subspecies is generally more cold tolerant than the *INDICA* subspecies, which is at least in part due to selection of specific transcription factor alleles, such as *bZIP73*, in the direct ancestor of *japonica* contributing to cold climate adaptation during early stages of rice domestication (Liu et al. 2018). Due to artificial human selection of other beneficial traits associated with warm climate adaptation, a trade-off between those traits and cold climate adaptation resulted in cold sensitivity of the *INDICA* subspecies, because there was no need to invest energy into genetic and metabolic pathways promoting cold tolerance.

Interestingly, we did not observe a good correlation between LTSS and EL at each chilling temperature, indicating that under certain low-temperature stress conditions, EL cannot be used to predict seedling survivability after stress recovery (Fig. 6). This lack of correlation might also suggest that at certain chilling temperatures, the degree of membrane damage sustained during stress exposure is not the only determinant for seedling survivability. That many data points deviated from the trendline even for the best EL-LTSS correlation at 8 °C suggests that there may be different cold tolerance response mechanisms controlling membrane integrity and survival in rice. For submergence stress, there are two major response mechanisms that rice uses to survive under hypoxia stress, including growth restriction and rapid growth and cell elongation (Septiningsih et al. 2009; Nagai et al. 2010; Miro and Ismail 2013). Analogous to this, there may be alternative or even competing stress tolerance response mechanisms that different cold tolerant accessions use under the same or varying low-temperature stress conditions.

### Genome-Wide Association Study (GWAS)-Based Mapping of EL, LTSS, and LT50 QTL

The 11 cold tolerance traits [(EL + LTSS) × 5 temperatures + LT50] we assessed yielded 245 quantitative trait loci (QTL) using 354 rice diversity panel 1 (RDP1) accessions comprising of all five major subpopulations of rice (*aromatic*, *aus*, *indica*, *temperate japonica*, *tropical japonica*) and some admixes between them. Because standard regression techniques used to perform association studies assume that all variables within a data set are mutually independent, genetic relatedness between individuals in a population can generate false positive associations (Sul et al. 2018). To correct for this, the publicly available GWAS pipeline we employed uses a mixed model based on EMMAX. We are, therefore, confident that the single nucleotide polymorphism (SNP) variants we identified as associated with the 11 cold tolerance traits are within a 95% confidence interval, because mixed models were previously shown in rice and humans to adequately correct for population structure in the sample (McCouch et al. 2016; Sul et al. 2018). Performing additional GWAS analyses using different subsets of accessions yielded another 255 QTL, many of which overlapped with the 245 QTL using all accessions. Together, the 500 QTL identified 178 unique chromosomal regions covering 25% of the rice genome, indicating that chilling tolerance in rice is a multigenic trait. Interestingly, 36 out of 39 (92%) seedling stage cold tolerance QTL we previously identified using the Rice Mini-Core collection (Schläppi et al. 2017) were validated by at least one QTL identified using RDP1 accessions, and 18 out of 39 (46%) were validated by multiple-trait QTL (*qMT*; Fig. 9; Table 1). Moreover, at least 23 out of 40 (57.5%) *qMT* QTL overlapped with legacy rice seedling stage cold tolerance QTL identified during the last 20 years (Fig. 9; Table 1). Taken together, the validation of many cold tolerance QTL that were previously identified using different collections of rice accessions suggests that the QTL shown here are robust and adequate for the identification of associated stress tolerance response genes.

### Chilling Stress-Relevant Gene Ontology (GO) Term Clusters

The GO term enrichment and clustering data suggested that the major GO term clusters for the genes found within the multiple-trait QTL have a high likelihood to be involved in different mechanisms of cold stress tolerance responses. In the clusters determined by the filtered list of 71 genes, some specific pathways emerged. “Golgi apparatus” as a significant cluster suggests an involvement of membrane transport from the endoplasmic reticulum (ER) to other locations in the cell. Together with GO term clusters derived from larger multiple-trait QTL genes, this suggests that the Golgi apparatus where ubiquitination determines the destination of different Golgi-associated proteins has an important function in regulating cold stress tolerance responses. It was previously shown that ubiquitination of proteins in the Golgi can be used as a signal for subcellular localization to many destinations, including, the nucleus, the vacuole, and the plasma membrane (Risinger and Kaiser 2008; Scheuring et al. 2012; Stone 2014; Liu and Li 2019). Ubiquitination and the Golgi apparatus are also involved in plant hormone signaling pathways (Stone 2014). These known interactions suggest that some of the candidate genes found in the filtered 71 gene list are involved in a Golgi-mediated cold stress response mechanism.

In addition to its intracellular localization function, the Golgi apparatus is involved in the secretory pathway for many cellular components. The GO enrichment in both the full multiple-trait QTL gene list and the filtered 71-gene list uncovered terms relevant to transmembrane transport. Specifically, the full-gene list enrichment resulted in term clusters of genes involved in both carbohydrate biosynthesis and carbohydrate transmembrane transport. This could be due to a difference in the ability of Tolerant and Sensitive clusters to manage osmotic homeostasis under chilling stress, an important component of chilling tolerance (Afzal et al. 2016). As the filtered gene list pointed to the involvement of the Golgi and ubiquitination, the pathways may converge in the process of subcellular localization or extracellular secretion of critical components for cell wall modification and long-range signaling (Park et al. 2017; Takahashi et al. 2019). These findings suggest that one of the chilling stress tolerance response mechanisms that is different between the *JAPONICA* and *INDICA* subspecies may be how the Golgi apparatus responds or functions during chilling stress.

A third class of candidate genes are associated with terms of signal transduction. Many GO term clusters or cluster members involved “nucleotide binding”, “signal”, and “protein modification by small molecules”. These terms suggest that differences in signal transduction pathways might explain cold tolerance differences between Tolerant and Sensitive accessions. These genes could be involved in transducing signals for phytohormones, mechanosensory channels, plasma membrane ion channels, or relaying secondary signaling events throughout the cell to plastids in response to chilling stress.

Finally, the term “regulation of plant growth and development” appeared multiple times in enrichments for different gene populations. The most obvious function for genes in this cluster is related to how plant growth and development is regulated during chilling stress. Analogous to the aforementioned submergence tolerance, one cold stress tolerance response mechanism might lead to complete growth cessation while another one might lead to cellular and metabolic homeostasis allowing a certain degree of growth and development even under cold temperatures. Interactions between growth-regulating genes and phytohormones are well known (Nagai et al. 2010; Colebrook et al. 2014; Sah et al. 2016), and hormones such as abscisic acid (ABA) have previously been linked to abiotic stress, including cold tolerance (Lado and Manzi 2017; Gibson 2004; Sah et al. 2016; Liu et al. 2018).

## Conclusions

This study provides novel insight into the potential mechanisms for cold tolerance in rice using GWAS-based mapping of two chilling tolerance traits under different low-temperature stress conditions. We show that at different chilling temperatures, different potential mechanisms might control cold stress tolerance responses to reduce plasma membrane damage and enhance seedling survivability. Interestingly, the highest (16 °C) and lowest (4 °C) temperatures tested had the least correlation between membrane damage and seedling survivability, suggesting differences in mechanisms involved in survival at these temperatures compared to 8–12 °C. The 500 QTL controlling membrane integrity and seedling survivability under chilling stress identified in this study cover approximately 25% of the rice genome, while the 40 multiple-trait QTL among them cover approximately 10% of the rice genome. This is in agreement with the notion that cold tolerance is a complex and polygenic trait. Genes within those genomic regions were enriched for terms involved in “carbohydrate biosynthesis and transmembrane transport”, “plasma membrane”, “ubiquitination”, and “ADP binding”. Among them, 71 genes with significant SNPs and allele effects were enriched for “Golgi apparatus”, “transmembrane transport”, “signal transduction”, and “stress response”. These enrichments suggest a likely involvement of the secretory and subcellular localization functions of the Golgi apparatus. With the introduction of QTL derived from cold tolerance cluster-specific mapping, the common themes of “plant growth”, “ubiquitination”, “signaling”, and “transmembrane transport” were highlighted. These terms, along with “Golgi apparatus”, suggest a difference between Tolerant and Sensitive accessions in the function of signaling pathways, possibly involving hormones such as ABA (Fig. S12). Investigation of candidate genes involved in or regulating these pathways is a critical next step in understanding the mechanistic differences in chilling stress tolerance responses between accessions from the *JAPONICA* and *INDICA* subspecies of rice.

## Methods

### Plant Materials

Seeds from 354 rice accessions were obtained from the USDA/ARS Genetics Stocks-*Oryza* (GSOR) collection. All of the accessions were all part of the Rice Diversity Panel 1 (RDP1) that were genotyped using the high-density rice array (HDRA) containing 700,000 (700 K) single nucleotide polymorphisms (SNPs; Eizenga et al. 2014; McCouch et al. 2016). These lines were used as a representation of the entire *Oryza sativa* species to map genotypic variances that explain the subspecies differences in chilling tolerance levels between *JAPONICA* and *INDICA*.

### Germination and Standard Growth Conditions

Seeds were germinated in tap water at 37 °C in the dark for 3 days in petri dishes with 0.1% bleach solution to prevent bacterial growth. Germinating seeds were moved to hydroponics boxes after 2 days of germination for the final day of germination to prevent root overgrowth and maintain the same direction of shoot growth. The hydroponics boxes were filled with deionized (DI) water with no bleach. After germination, the hydroponics boxes were moved to a chamber with standard growth conditions of 12 h light/12 h dark cycles and 28 °C/25 °C day/night temperatures. The germinated seeds were allowed to grow until 2-weeks of age. At 10 days post germination, the water in the boxes were replaced with ¼-strength Murashige-Skoog basal salt medium (Murashige and Skoog 1962). Each accession was represented by up to 8 plants per box in a triplicate randomized block design for up to 24 plants (3 boxes) per experiment.

### Chilling Stress Treatment

Two-week-old, seedlings were transferred for 7 days to a low-temperature growth chamber with a 12 h/12 h light/dark cycle and a constant chilling temperature. Watering was adjusted to a minimum due to significantly lower water depletion in boxes and an effort to minimize chamber temperature fluctuations. Five chilling temperatures were tested independently: 4 °C, 8 °C, 10 °C, 12 °C, and 16 °C. At the end of the 7-day period, leaf samples were taken for electrolyte leakage (EL) measurements and plants were returned to standard growth conditions (12 h light/12 h dark, 28 °C/25 °C day/night cycles) after replacement of hydroponic solution with fresh ¼ x MS media.

### Recovery

Plants were allowed to recover for 7 days in normal growth conditions, with watering at least every other day. Low-temperature seedling survivability (LTSS) was recorded, as described below, on day 7.

### EL and LTSS Chilling Tolerance Trait Phenotyping

EL was measured after 7 days of chilling stress treatment. For each cultivar, tissue segments were cut from the center of four leaves, washed in DI water, and pooled into glass screw-cap tubes containing 5 mL of DI water having at a conductivity of no more than 1 μS/cm. The tubes were shaken for 1 h and initial conductivity was measured twice using a LAQUA twin B-771 hand-held conductivity meter (HORIBA Scientific, Japan). The tubes were then boiled for 10 min, shaken for 10 min, and allowed to cool. The total conductivity was measured from cooled tubes. Mean percent EL was then calculated as %EL = [(initial EL divided by total EL) times 100]. Replicate EL values were averaged, and that average was used for quantitative trait loci (QTL) mapping.

LTSS was measured after 7 days of recovery. Alive and dead seedlings were determined by visual inspection as previously described (Schläppi et al. 2017): alive were green and healthy looking; dead were bleached and wilted. The mean percent survivability was calculated as %LTSS = [(number of green and healthy-looking seedlings after 1 week of recovery growth divided by the number of initial healthy-looking seedlings before treatment) times 100]. Replicate LTSS values were averaged, and that average was used for QTL mapping.

Median lethal temperature (LT50) was calculated using dead and alive counts from LTSS recordings of all five temperatures analyzed. LTSS curves as a function of temperature were calculated using a generalized linear model using a binomial distribution in *R* (R Core Team 2018). The *R* script used was derived from https://lukemiller.org/index.php/2010/02/calculating-lt50-median-lethal-temperature-aka-ld50-quickly-in-r/ (Miller 2010).

### Genome-Wide Association Study (GWAS)-Based QTL Mapping

Cold stress tolerance phenotyping was done on a subset of 354 out of 424 RDP1 accessions that were used for genotyping with the 700 K SNPs of the HDRA (Chen et al. 2014; McCouch et al. 2016). A GWAS mapping python pipeline for this subset of RDP1, created by McCouch et al. 2016, was used for GWAS-based QTL mapping and generating Manhattan and quantile-quantile (Q-Q) plots. The pipeline uses a variance component approach implemented in the publicly available software, efficient (linear) mixed-model association eXpedited (EMMAX; Kang et al. 2010), to map HDRA-derived 700 K SNPs to phenotype and to account for population structure by calculating and including a kinship matrix as a covariate, which helps to distinguish true associations from false positives due to relatedness between accessions. The kinship matrix was calculated using identity by state (IBS) to group identical allele states while excluding shared ancestry. The default allele filtering settings were used to run the pipeline: minor allele frequency (MAF) > 10%, minor allele count (MAC) > 1, percent missing allele < 30%.

### Quantitative Trait Loci (QTL)

QTL were identified using a protocol similar to one used by Shakiba et al. 2017. SNPs were determined significant if they had -log10(*p*) > 4, because most of the Q-Q plots diverged from expected near 4 (Fig. S1). QTL were assigned when 3 or more significant SNPs were found within a 1 Mb region. Eleven phenotypes were used for GWAS-based QTL mapping: 5 sets of EL data from 5 temperatures tested, 5 sets of LTSS data from 5 temperatures tested, and the calculated LT50 for each of the 354 accessions.

### Candidate Gene Identification and Gene Ontology (GO) Term Analysis

Genes within QTL were identified using the Allele Finder web tool provided by the Rice Diversity Project (McCouch et al. 2016). All genes found within a QTL were deemed potential candidate genes. GO term enrichments of all genes identified within QTL regions were determined using the GO Enrichment Analysis web tool from http://plantregmap.cbi.pku.edu.cn. GO term clustering and enrichment visualization was done by using the REVIGO webtool from http://revigo.irb.hr (Supek et al. 2011).

### Candidate Gene Filtering

Genes were filtered based on expected mechanistic involvement in chilling tolerance due to GO term annotation as well as tissue and stress responsive expression data provided by the Rice Genome Annotation Project from Michigan State University (Kawahara et al. 2013). Using gene descriptions, transposable elements were filtered out to focus the search on protein encoding genes likely to be involved in chilling tolerance responses. GO enrichment analysis was conducted on these genes to identify likely functional enrichment as a guide to understanding possible cold tolerance mechanisms. The transposon-free list of genes was also filtered based on the presence of significant SNPs identified by GWAS mapping to find genes with SNP variations that resulted in significant effects on chilling tolerance traits. These genes were further filtered to identify SNPs with major allele distribution relatively close to the proportion of accessions belonging to the Tolerant cluster (205 out of 354 accessions = 58%) to identify genes involved in mechanisms underlying cluster-specific chilling tolerance phenotypes.

### Statistical Analysis

Significant differences for all cold tolerance phenotypes were calculated using an *R* computation of Welch’s ANOVA and a Games-Howell non-parametric post hoc test due to significant differences in sample size as well as violation of the assumption of normality when comparing data between accession clusters.

## Supplementary information

**Additional file 1 Figure S1.** Quantile-Quantile (Q-Q) plots for GWAS mapping.

**Additional file 2 Figure S2.** Approximate genomic locations of 40 Multiple-Trait (*qMT*) QTL.

**Additional file 3 Figure S3.** Multiple-Trait QTL gene Biological Processes enrichment map.

**Additional file 4 Figure S4.** Multiple-Trait QTL gene Cell Component enrichment map.

**Additional file 5 Figure S5.** Multiple-Trait QTL gene Molecular Function enrichment map.

**Additional file 6 Figure S6.** Filtered Significant SNP Biological Processes enrichment map.

**Additional file 7 Figure S7.** Filtered Significant SNP Cell Component enrichment map.

**Additional file 8 Figure S8.** Filtered Significant SNP Molecular Function enrichment map.

**Additional file 9 Figure S9.** Multiple-Trait+Cluster QTL Biological Process enrichment map.

**Additional file 10 Figure S10.** Multiple-Trait+Cluster QTL Cell Component enrichment map.

**Additional file 11 Figure S11.** Multiple-Trait+Cluster QTL Molecular Function enrichment map.

**Additional file 12 Figure S12:** Model for functional associations of candidate term clusters.

**Additional file 13 Tables S1-S10.** QTL positions resulting from various GWAS mappings.

## Data Availability

All data generated or analyzed during this study are included in this article and its supplementary information files.

## Abbreviations

ABA: Abscisic acid
EL: Electrolyte leakage
EMMAX: Efficient mixed-model association eXpedited
GWAS: Genome-wide association study/ies
HDRA: High density rice array
IBS: Identity by state
LT50: Median lethal chilling temperature
LTSS: Low temperature seedling survivability
Mbp: One million base pair
Q-Q: Quantile-quantile
QTL: Quantitative trait locus/i
*qE*: EL QTL
*qL*: LTSS QTL
*qMT*: Multiple-trait QTL
RDP1: Rice diversity panel 1
SNP: Single nucleotide polymorphism(s)
TvS: Tolerant versus Sensitive
