# Supplementary material for: Multiple Cold Tolerance Trait Phenotyping Reveals Shared Quantitative Trait Loci in Oryza sativa
Source: Rice (N Y). 2020 Aug 14;13:57. doi: 10.1186/s12284-020-00414-3 (PMC7427827; doi:10.1186/s12284-020-00414-3)
Supplement: Supplementary file 1 — Additional file 1 Figure S1. Quantile-Quantile (Q-Q) plots for GWAS mapping. [file 12284_2020_414_MOESM1_ESM.docx]

**Supplementary Fig. S1** Quantile-Quantile (Q-Q) plots for all cold tolerance trait GWAS mapping. Plots were created using the RDP1 GWAS mapping pipeline (McCouch et al. 2016). Divergence of SNPs from expected (significance cutoff) is apparent at -log10(*p*)> 4 for all plots.
