# Supplementary material for: Multiple Cold Tolerance Trait Phenotyping Reveals Shared Quantitative Trait Loci in Oryza sativa
Source: Rice (N Y). 2020 Aug 14;13:57. doi: 10.1186/s12284-020-00414-3 (PMC7427827; doi:10.1186/s12284-020-00414-3)
Supplement: Supplementary file 2 — Additional file 2 Figure S2. Approximate genomic locations of 40 Multiple-Trait (qMT) QTL. [file 12284_2020_414_MOESM2_ESM.docx]

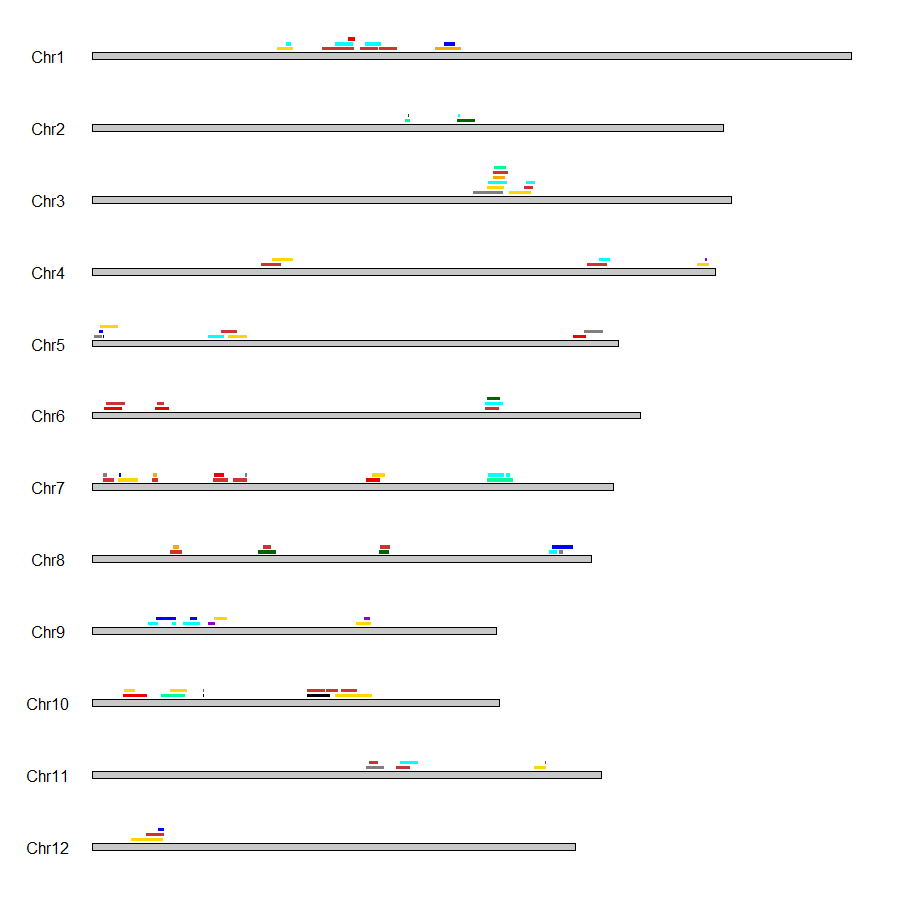


**Supplementary Fig. S2** Approximate genomic locations of 40 Multiple-Trait (*qMT*) QTL. Colored bars show the approximate lengths of Individual Cold Tolerance QTL for 11 traits: Electrolyte Leakage (EL) measured at 4°C, 8°C, 10°C, 12°C, and 16°C (Fig. 4); Low-Temperature Seedling Survivability (LTSS) measured at 4°C, 8°C, 10°C, 12°C, and 16°C (Fig. 5); and Median Lethal Temperature (LT50, Fig. 7). Exact genomic locations are shown in Table 1 and Fig. 9.
