# Supplementary material for: Multiple Cold Tolerance Trait Phenotyping Reveals Shared Quantitative Trait Loci in Oryza sativa
Source: Rice (N Y). 2020 Aug 14;13:57. doi: 10.1186/s12284-020-00414-3 (PMC7427827; doi:10.1186/s12284-020-00414-3)
Supplement: Supplementary file 3 — Additional file 3 Figure S3. Multiple-Trait QTL gene Biological Processes enrichment map. [file 12284_2020_414_MOESM3_ESM.docx]

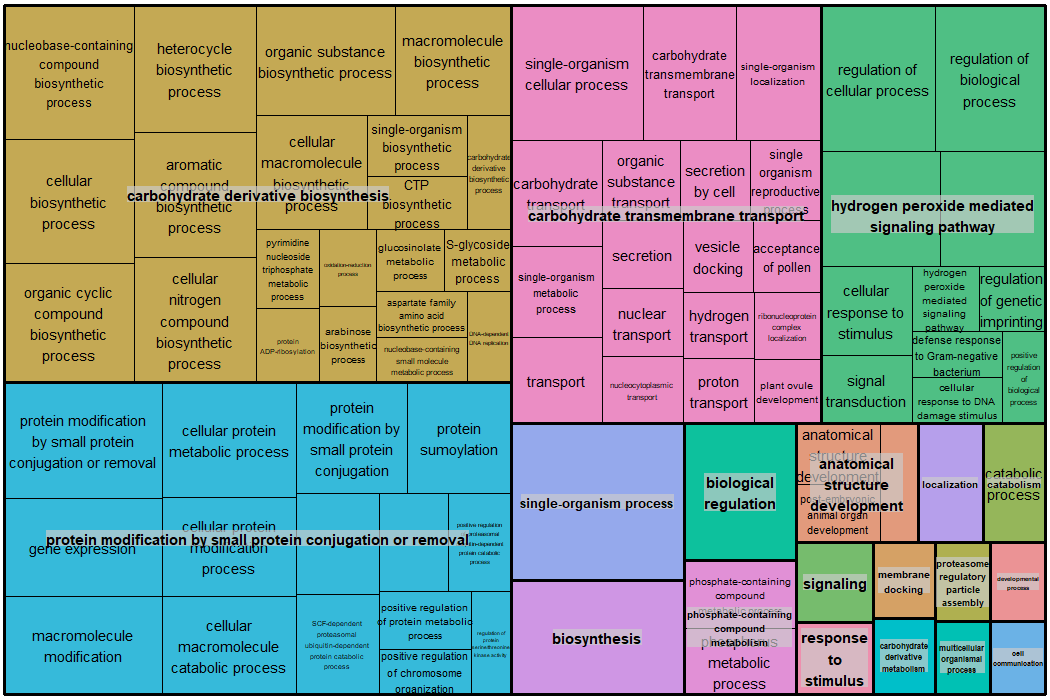


**Supplementary Fig. S3** Multiple Trait QTL (*qMT*) *Biological Processes* enrichment tree map. GO term enrichment analysis of all genes within *qMT* QTL is shown. GO term similarity was calculated using simRel scores, and a tree map for Biological Processes was constructed using REVIGO.
