# Supplementary material for: Multiple Cold Tolerance Trait Phenotyping Reveals Shared Quantitative Trait Loci in Oryza sativa
Source: Rice (N Y). 2020 Aug 14;13:57. doi: 10.1186/s12284-020-00414-3 (PMC7427827; doi:10.1186/s12284-020-00414-3)
Supplement: Supplementary file 4 — Additional file 4 Figure S4. Multiple-Trait QTL gene Cell Component enrichment map. [file 12284_2020_414_MOESM4_ESM.docx]

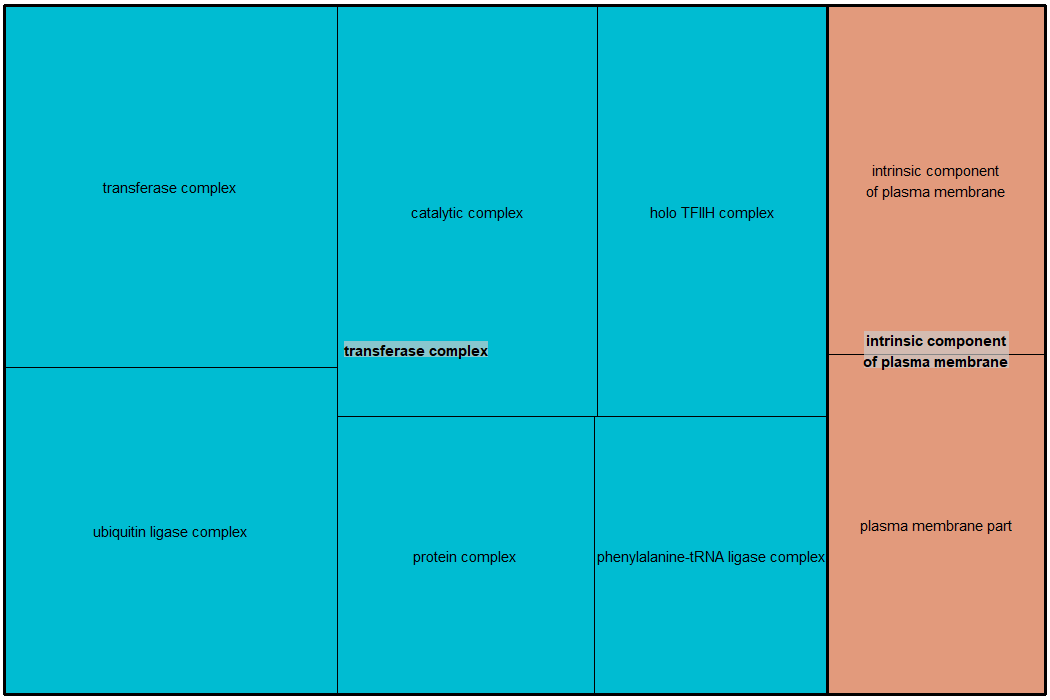


**Supplementary Fig. S4** Multiple Trait QTL (*qMT*) *Cell Component* enrichment tree map. GO term enrichment analysis of all genes within *qMT* QTL is shown. GO term similarity was calculated using simRel scores, and a tree map for Biological Processes was constructed using REVIGO.
