# Supplementary material for: Multiple Cold Tolerance Trait Phenotyping Reveals Shared Quantitative Trait Loci in Oryza sativa
Source: Rice (N Y). 2020 Aug 14;13:57. doi: 10.1186/s12284-020-00414-3 (PMC7427827; doi:10.1186/s12284-020-00414-3)
Supplement: Supplementary file 6 — Additional file 6 Figure S6. Filtered Significant SNP Biological Processes enrichment map. [file 12284_2020_414_MOESM6_ESM.docx]

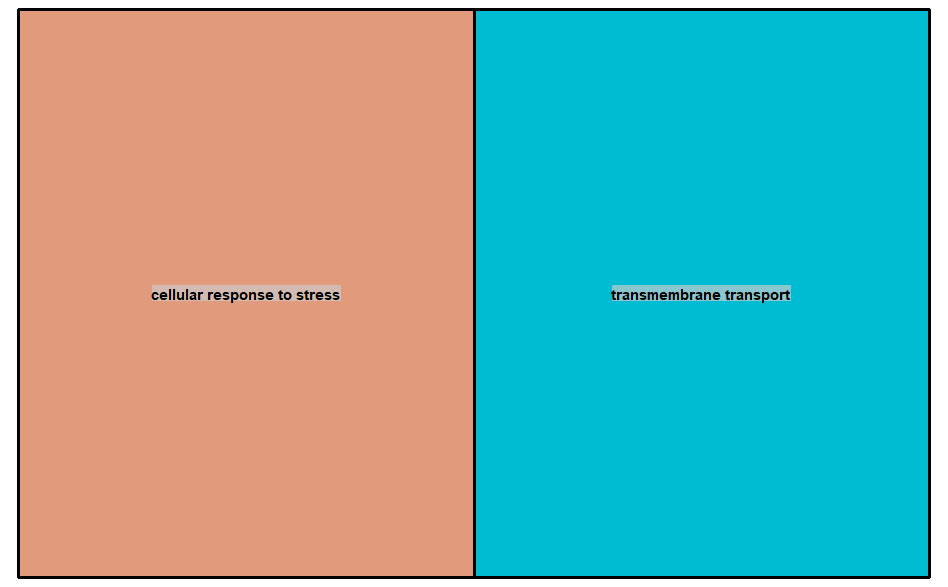


**Supplementary Fig. S6** Filtered Gene List *Biological Processes* enrichment tree map. GO term enrichment analysis for 71 filtered genes within *qMT* QTL is shown. GO term similarity was calculated by simRel scores and a tree map for Biological Processes was constructed by REVIGO.
