# Supplementary material for: Multiple Cold Tolerance Trait Phenotyping Reveals Shared Quantitative Trait Loci in Oryza sativa
Source: Rice (N Y). 2020 Aug 14;13:57. doi: 10.1186/s12284-020-00414-3 (PMC7427827; doi:10.1186/s12284-020-00414-3)
Supplement: Supplementary file 8 — Additional file 8 Figure S8. Filtered Significant SNP Molecular Function enrichment map. [file 12284_2020_414_MOESM8_ESM.docx]

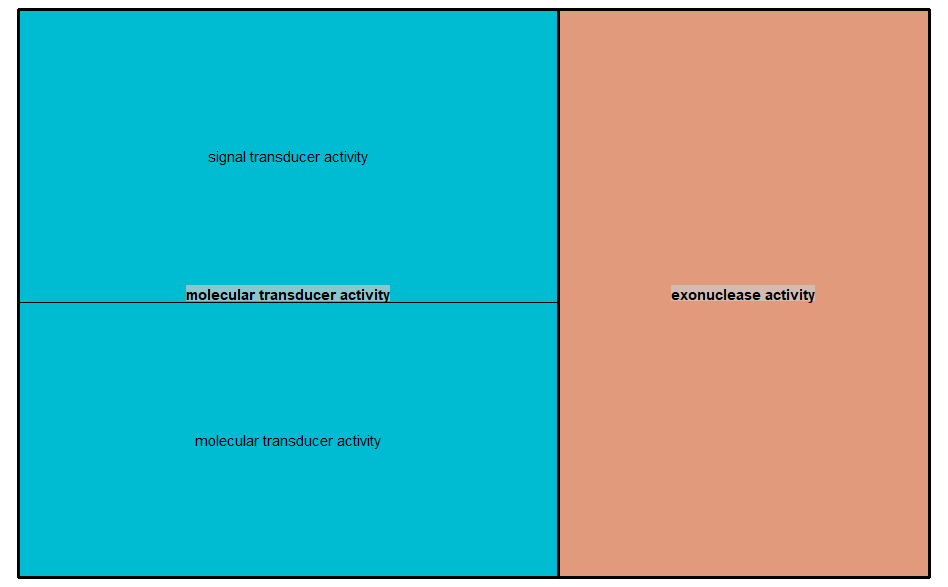


**Supplementary Fig. S8** Filtered Gene List *Molecular Function* enrichment tree map. GO term enrichment analysis for 71 filtered genes within *qMT* QTL is shown. GO term similarity was calculated by simRel scores and a tree map for Molecular Function was constructed by REVIGO.
