# Supplementary material for: Multiple Cold Tolerance Trait Phenotyping Reveals Shared Quantitative Trait Loci in Oryza sativa
Source: Rice (N Y). 2020 Aug 14;13:57. doi: 10.1186/s12284-020-00414-3 (PMC7427827; doi:10.1186/s12284-020-00414-3)
Supplement: Supplementary file 9 — Additional file 9 Figure S9. Multiple-Trait+Cluster QTL Biological Process enrichment map. [file 12284_2020_414_MOESM9_ESM.docx]

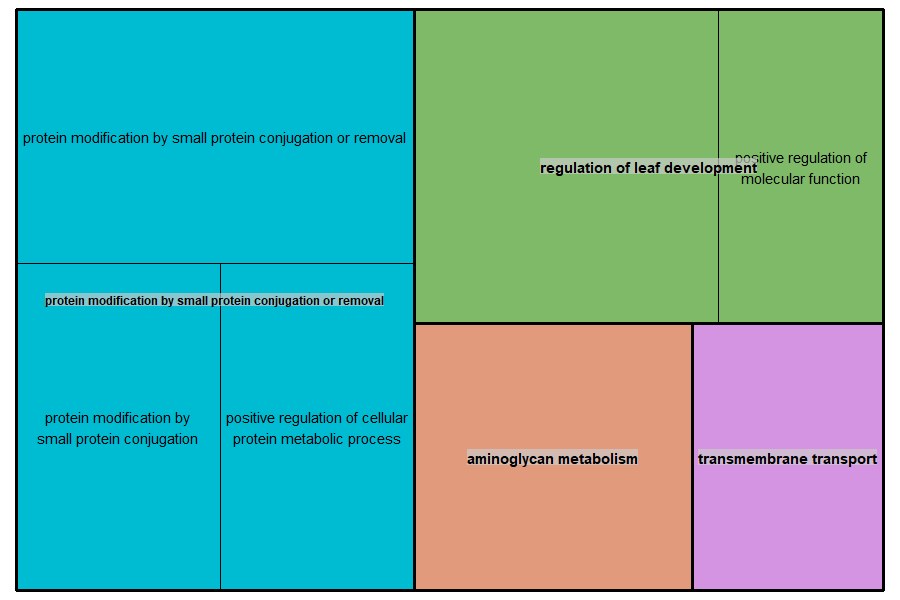


**Supplementary Fig. S9** Multiple Trait (*qMT*) QTL + Tolerant & Sensitive Cluster QTL *Biological Process* enrichment map. GO term enrichment analysis of 159 filtered genes within *qMT* + cluster specific QTL is shown. GO term similarity was calculated by simRel scores and a tree map for Molecular Function was constructed by REVIGO.
