# Supplementary material for: Multiple Cold Tolerance Trait Phenotyping Reveals Shared Quantitative Trait Loci in Oryza sativa
Source: Rice (N Y). 2020 Aug 14;13:57. doi: 10.1186/s12284-020-00414-3 (PMC7427827; doi:10.1186/s12284-020-00414-3)
Supplement: Supplementary file 12 — Additional file 12 Figure S12: Model for functional associations of candidate term clusters. [file 12284_2020_414_MOESM12_ESM.docx]

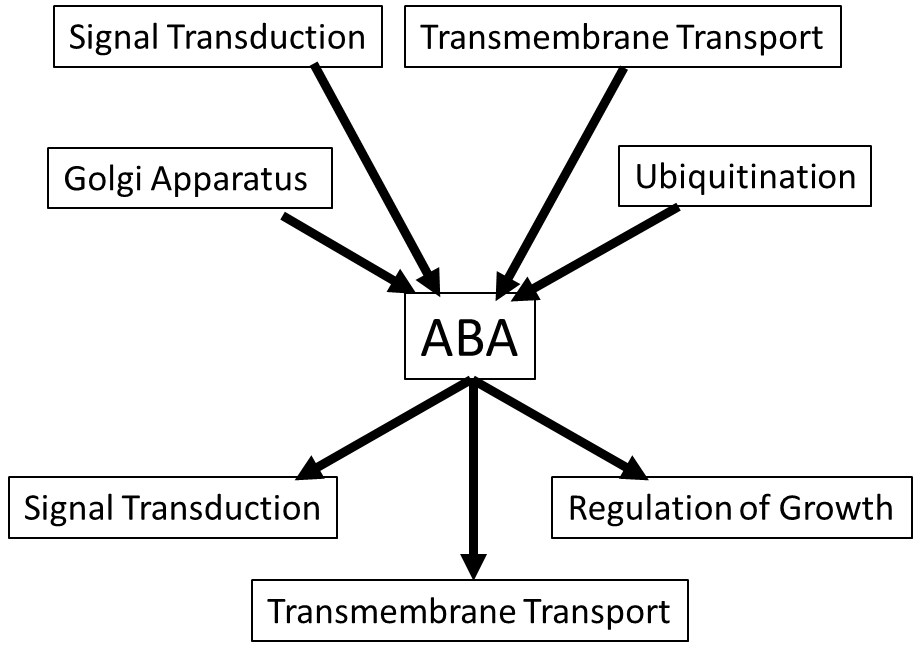


**Supplementary Fig. S12** Model for functional associations of candidate term clusters. Shown is one possibility of how candidate genes from different GO term clusters may control cold tolerance through the ABA signaling pathway. Alternative models are possible (not shown).
